# Research hotspots and trends of epigenetic therapy in oncology: a bibliometric analysis from 2004 to 2023

**DOI:** 10.3389/fphar.2024.1465954

**Published:** 2024-09-12

**Authors:** Sisi Li, Xinrui Liang, Qing Shao, Guanwen Wang, Yuxin Huang, Ping Wen, Dongping Jiang, Xiaohua Zeng

**Affiliations:** ^1^ Department of Breast Cancer Center, Chongqing University Cancer Hospital, Chongqing, China; ^2^ Chongqing Key Laboratory for Intelligent Oncology in Breast Cancer (iCQBC), Chongqing University Cancer Hospital, Chongqing, China; ^3^ School of Medicine, Chongqing University, Chongqing, China

**Keywords:** cancer, epigenetic, therapy, bibliometric, VOSviewer, Citespace

## Abstract

**Background:**

Epigenetics denotes heritable alterations in gene expression patterns independent of changes in DNA sequence. Epigenetic therapy seeks to reprogram malignant cells to a normal phenotype and has been extensively investigated in oncology. This study conducts a bibliometric analysis of epigenetic therapy in cancer, providing a comprehensive overview of current research, identifying trends, and highlighting key areas of investigation.

**Methods:**

Publications concerning epigenetic inhibitors in cancer spanning 2004 to 2023 were retrieved from the Web of Science Core Collection (WoSCC). Co-occurrence analysis using VOSviewer assessed current status and focal points. Evolutionary trends and bursts in the knowledge domain were analyzed using CiteSpace. Bibliometrix facilitated topic evolution and revealed trends in keywords. National, institutional, and author affiliations and collaborations were also examined.

**Results:**

A total of 2,153 articles and reviews on epigenetic therapy in oncology were identified, demonstrating a consistent upward trend over time. The United States (745 papers), University of Texas MD Anderson Cancer Center (57 papers), and Stephen B. Baylin (27 papers) emerged as the most productive country, institution, and author, respectively. Keyword co-occurrence analysis identified five primary clusters: tumor, DNA methylation, epigenetic therapy, expression, and immunotherapy. In the past 5 years, newly emerging themes with increased centrality and density include “drug resistance,” “immunotherapy,” and “combination therapy.” The most cited publication reviewed current understanding of potential causes of epigenetic diseases and proposed future therapeutic strategies.

**Conclusion:**

In the past two decades, the importance of epigenetic therapy in cancer research has become increasingly prominent. The United States occupies a key position in this field, while China, despite having published a large number of related papers, still has relatively limited influence. Current research focuses on the “combination therapy” of epigenetic drugs. Future studies should further explore the sequencing and scheduling of combination therapies, optimize trial designs and dosing regimens to improve clinical efficacy.

## 1 Introduction

The term “epigenetics” was originally coined by C.H. Waddington (Waddington, 2012) to describe heritable changes in cellular phenotype independent of alterations in DNA sequence (Dawson and Kouzarides, 2012). Epigenetic modifications convey regulatory information crucial in all DNA-based processes such as transcription, DNA repair, and replication (Kouzarides, 2007). Aberrant expression patterns in chromatin regulators or genomic alterations can profoundly influence the initiation and maintenance of various cancers (Dawson and Kouzarides, 2012). Promoter hypermethylation and global hypomethylation have been observed in cancers, contributing respectively to transcriptional silencing and genomic instability (Jones and Baylin, 2002; Eden et al., 2003; Rodriguez et al., 2006).

DNA methylation, histone modifications, nucleosome remodeling, and RNA-mediated targeted regulation are critical biological processes underlying cancer pathogenesis (Dawson and Kouzarides, 2012). Global DNA hypomethylation is closely associated with chromosomal rearrangements and nuclear disorganization in cancer cells, leading to chromosomal instability (Hoffmann and Schulz, 2005). For instance, follicular lymphoma demonstrates recurrent mutations in the histone methyltransferase MLL2 in nearly 90% of cases (Morin et al., 2011). Similarly, UTX, a histone demethylase, is mutated in up to 12 histologically distinct cancers (van Haaften et al., 2009). Genetic alterations in chromatin modifiers and global changes in the epigenetic landscape not only underscore their pathological roles in oncology but also highlight potential therapeutic targets for intervention (Dawson and Kouzarides, 2012).

The therapeutic potential of epigenetic therapies lies in their ability to reverse epigenetic changes, unlike genetic abnormalities, thereby restoring normal gene function affected by these alterations (Baylin and Jones, 2011; Ahuja et al., 2014). Current epigenetic therapies primarily involve DNA demethylation and histone deacetylase inhibitors [12]. While the former is FDA-approved for myelodysplastic syndromes (MDS), histone deacetylase (HDAC) inhibitors have gained FDA-approved for T-cell cutaneous lymphoma and multiple myeloma (Kaminskas et al., 2005; Azad et al., 2013). More and more studies are dedicated to exploring the effectiveness of epigenetic inhibitor in the treatment of solid tumors. Furthermore, numerous drugs targeting epigenetic regulation are under development and entering clinical trial stages (Ahuja et al., 2016).

However, despite the encouraging results of epigenetic inhibitors in the treatment of acute myeloid leukemia (AML), MDS, and chronic myeloid leukemia (CML), the efficacy of first-generation epigenetic drugs in patients with solid tumors has been disappointing (Cheng et al., 2019). Compared to hematologic malignancies, solid tumors are at a disadvantage due to their genomic complexity, drug exposure environment, and tumor heterogeneity (Yang et al., 2023). Preclinical studies and clinical trials have shown that combining epigenetic drugs with other therapies (such as chemotherapy, targeted therapy, or immunotherapy) may provide the best opportunity to enhance clinical responses in solid tumors (Feng and De Carvalho, 2022). Therefore, further elucidating the progress, trends, and focal points in the field of epigenetic therapy is crucial for researchers engaged in related studies.

Bibliometric analysis, a popular and rigorous method, explores and analyzes scientific research outcomes and trends to identify data correlations. As a systematic analytical technique, bibliometrics can provide valuable insights for future researchers, helping them track hotspots and trends (Danthi et al., 2014), and forecast reports on the future development of specific research fields (Hicks et al., 2015). To date, there has been no bibliometric analysis focusing on epigenetic therapies in cancer treatment. Therefore, this study offers a thorough visual and bibliometric analysis of epigenetic therapies in oncology, identifying current trends and future directions in their application.

## 2 Materials and methods

### 2.1 Searching strategy and data collection

**FIGURE 1:**
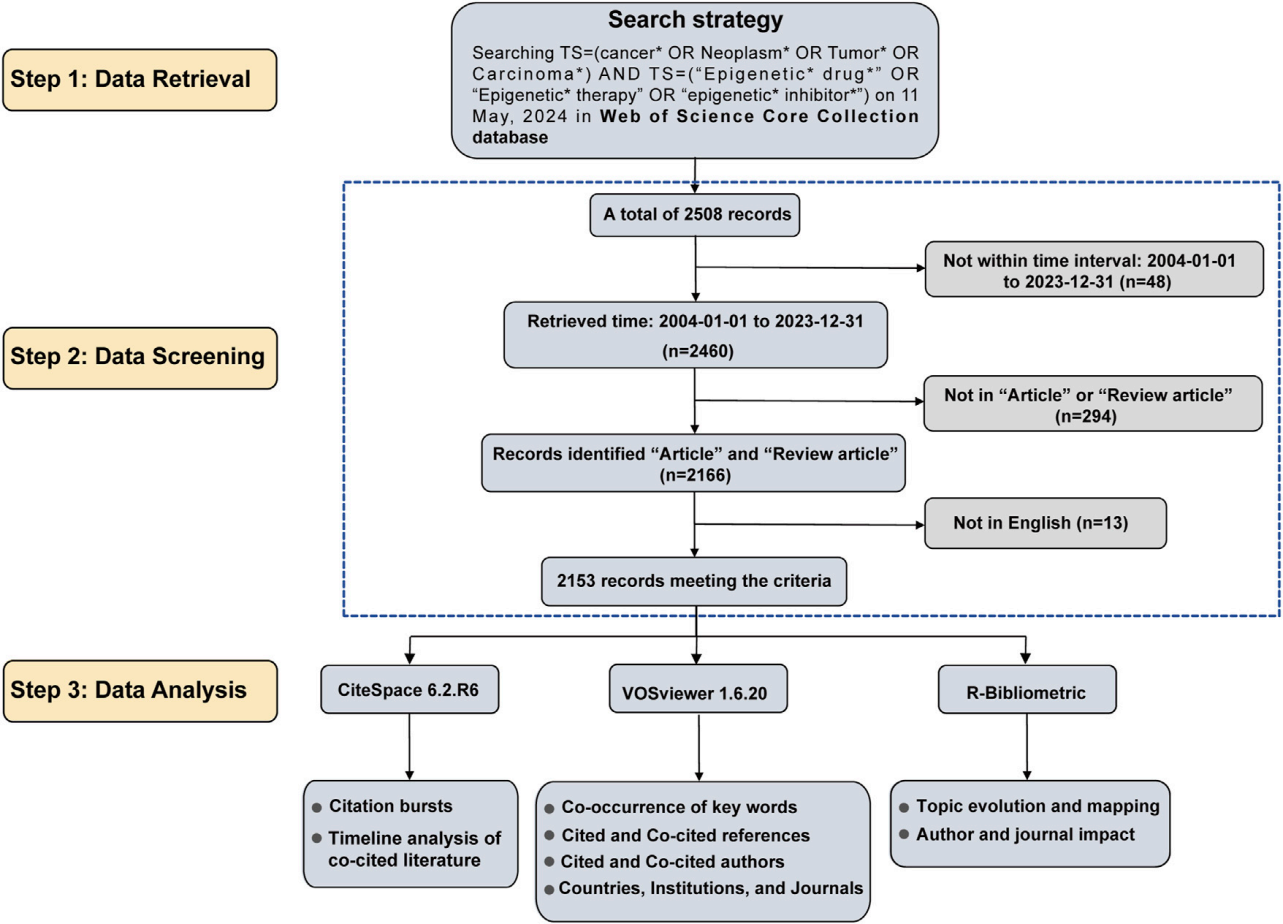
The flowchart of searching and selection process.

The original data for this study was obtained from the largest and most authoritative database, the Web of Science Core Collection (WoSCC) (Merigó and Núñez, 2016). Two researchers conducted independent searches, restricting the publication dates to 1 January 2004, through 31 December 2023. The search query used was as follows: Keywords [TS=(cancer* OR Neoplasm* OR Tumor* OR Carcinoma*) AND TS=(“Epigenetic* drug*” OR “Epigenetic* therapy” OR “epigenetic* inhibitor*”)]. The search was performed on 11 June 2024, yielding a total of 2,508 articles. First, 48 articles that fell outside the date range of 1 January 2004, to 31 December 2023, were excluded. Second, the document types were limited to “Article” and “Review,” resulting in the exclusion of 294 conference papers, commentaries, editorials, and other publications. Additionally, due to a restriction to English language only, 13 articles were excluded. After the screening process, a total of 2,153 papers were included. The data was exported in plain text format and labeled “download.” The data filtering process is illustrated in Figure 1.

### 2.2 Data analysis and mapping

The retrieved data was imported into Citespace (version 6.2.R6), VOSviewer (version 1.6.20), and the bibliomearch package (version 3.2.1) of R (4.3.0, https://www.r-project.org/) to visualize co-authorship networks, institutions, authors, journals, keywords, and co-citation networks of the articles.

VOSviewer, a widely recognized literature analysis software, visually illustrates scientific research trends within a specific field based on relationships among terms in academic literature, including authors, journals, and keywords. Analysis units in VOSviewer encompass countries, journals, authors, and keywords, depending on the analytical focus and database type. In this investigation, VOSviewer was employed for co-citation analysis, co-authorship analysis, and co-occurrence analysis. Co-citation analysis refers to the instances where two articles are cited together by a third article, indicating a citation relationship between the two (Ahmad and Slots, 2021). Co-authorship analysis reveals scientific collaborations, identifying cases where different authors, institutions, or countries/regions coexist in publications (Wu et al., 2021). Co-occurrence signifies the occurrence of two keywords within the same paper. Each node on the VOSviewer map corresponds to a specific parameter, such as authors, institutions, or countries. Node size indicates the number of publications, citation counts, or frequency of occurrence. Colors are assigned to clusters to categorize nodes and lines. Lines connecting nodes represent relationships between them (Xie et al., 2020).

CiteSpace, a Java-based software tool designed by Professor Chaomei Chen, is widely recognized for its utility in visualizing bibliometric characteristics and forecasting research trends within academic fields (Chen, 2004). In our study aimed at unraveling the knowledge base and evolution of this particular field, we leveraged CiteSpace for timeline analysis and detection of citation bursts within co-cited literature. “Citation bursts” denote sudden surges in the frequency of citations of a specific nature or a significant number of citations occurring within a defined timeframe. The term “Strength” indicates the intensity of the burst, “Start” signifies the initial year of the burst, and “End” denotes its termination. The presence of red bars on the timeline signifies the duration of the burst, while blue bars represent citations spanning the period from 2004 to 2024 (Zhao et al., 2024). Additionally, the parameters used in CiteSpace included: 1) Time span ranging from 2004 to 2024; 2) Slice duration of 1 year per slice; 3) Enabled pruning options such as pathfinder, minimum spanning tree, pruning slices network, and pruning merged network; 4) Top N set to 50; 5) All remaining parameters maintained at default values.

Bibliometrix, an R-based tool, is specifically designed to construct comprehensive scientific maps of published literature (Aria and Cuccurullo, 2017). The process of topic evolution and mapping entails clustering topics based on keywords found in publications and then mapping them to low-dimensional space to depict trends in topic changes. Topic evolution is visually represented through Sankey diagrams, which effectively showcase the shifts in topics across various time slices. In these diagrams, topic maps utilize density indices along the *y*-axis and centrality indices along the *x*-axis. Density signifies the strength of internal connections among keywords within a particular topic, while centrality reflects the strength of connections between the topic and other external topics. These maps are segmented into four quadrants: Q1 denotes core topics, indicating significant and well-developed themes; Q2 represents niche topics, which are highly developed but less interconnected with other themes; Q3 signifies emerging or declining topics, characterized by lower internal and external connections that indicate emerging or declining trends; and Q4 encompasses basic topics that are considered fundamental and cross-sectional in the field (Cobo et al., 2011) Changes and trajectories across distinct time periods are identified to discern the emergence or decline of topics. Within this domain, productivity quantification metrics are widely employed, including author and journal impact indices such as the H-index (Hirsch, 2007) and G-index (Egghe, 2006).

All original data used in this study were sourced from publicly available databases and did not involve participant information; therefore, no ethical review was deemed necessary.

## 3 Results

### 3.1 Analysis of publication output

**FIGURE 2:**
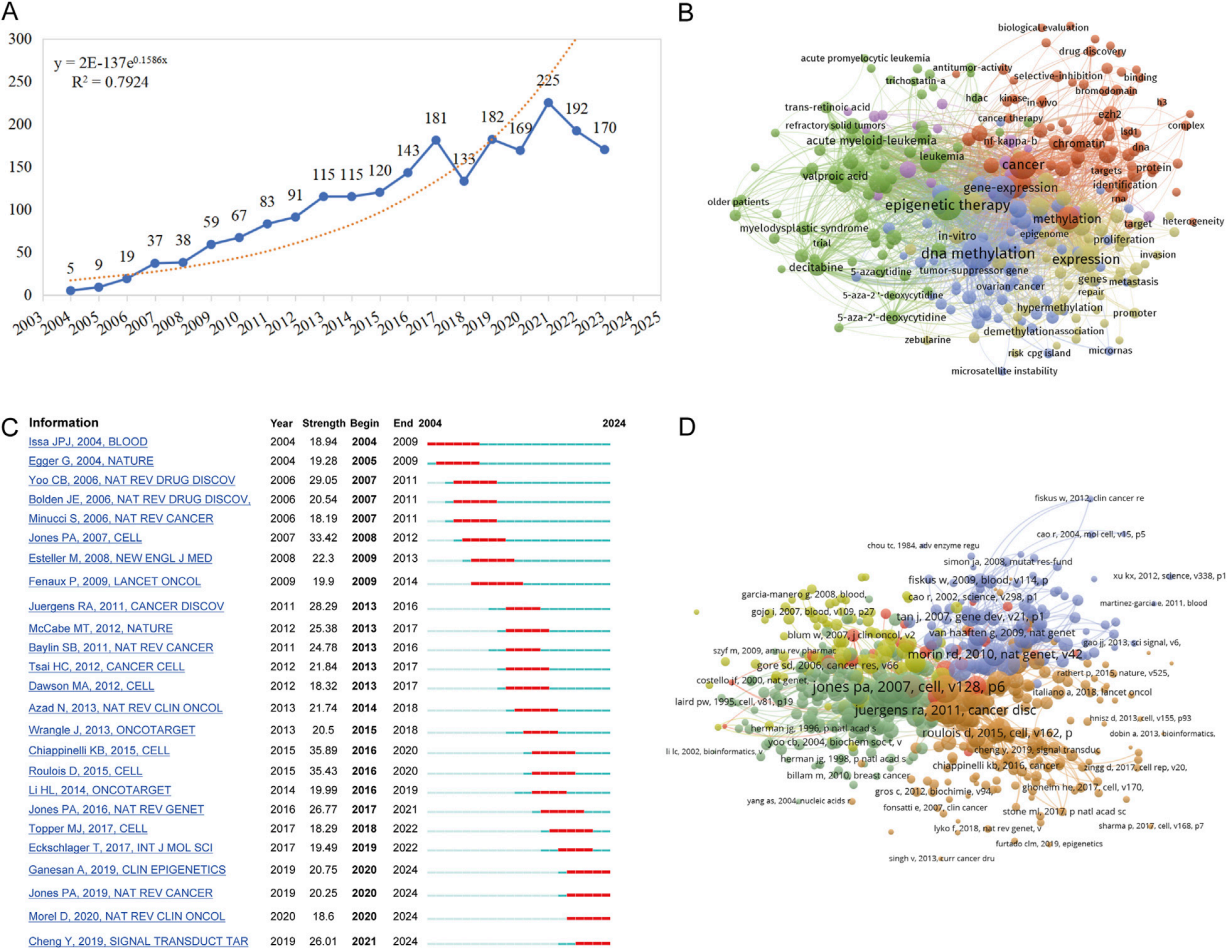
Hotspots and Bursts of Co-Citation References in Epigenetic Therapy Research within Oncology. **(A)** Trends in the global publication numbers over time. **(B)** Co-occurrence analysis of keywords. Node sizes correspond to keyword frequency, while node colors indicate their category in cluster analysis. **(C)** Top 25 references demonstrating strong citation bursts. A burst signifies a notable increase in citation frequency for a specific article. The red bar denotes the period when the reference co-citation burst commenced. **(D)** The network visualization of the relationship of co-cited reference.

Between 1 January 2004, and 31 December 2023, a total of 2,460 publications pertaining to tumor epigenetic therapy research were identified, including 2,166 articles and reviews, accounting for 32.5% of the total. Figure 1 illustrates the flowchart detailing the literature search and selection process, which facilitated the exclusion of irrelevant publications, ultimately leading to the analysis of 2,153 articles. Over this timeframe, the number of studies focusing on tumor epigenetic therapy surged from 5 in 2004 to 170 in 2023, marking a remarkable 33-fold increase (Figure 2A). The years spanning from 2004 to 2013 were characterized by an initial phase of development, witnessing a steady rise in the number of publications within this domain. Subsequently, from 2014 to 2017, a phase of rapid advancement in publications related to epigenetic therapy in oncology emerged. Despite minor fluctuations in publication numbers during 2018 and 2023, the cumulative total stood at 2,153 publications over the two-decade period, averaging approximately 107.65 publications annually. The collective citation count for all publications amounted to 283,310, with an average of 35.52 citations per publication.

### 3.2 Hotspots of keywords

To comprehend the core content and prevalent themes within this area of study, we conducted an analysis of author’s keywords extracted from the literature search. Table 1 illustrates the top 20 most frequently appearing author’s keywords. Key research domains in this field include DNA methylation, histone deacetylase inhibitor(s), decitabine, epigenetic drugs, HDAC inhibitors, among others. Leveraging these author’s keywords, we performed a keyword co-occurrence analysis using VOSviewer, as depicted in Figure 2B. A total of 285 keywords were identified with a usage frequency exceeding 15 instances, forming 5 distinct clusters (Figure 2B; Supplementary Material 1). The orange cluster (cluster 1) predominantly revolves around cancer, with studies focusing on aspects such as methylation, chromatin, histone deacetylase, acetylation, and DNA methyltransferase. The green cluster (cluster 2) encapsulates methodologies of epigenetic therapy, including histone deacetylase inhibitors, HDAC inhibitors, suberoylanilide hydroxamic acid, decitabine, DNA methyltransferase inhibitors, 5-azacytidine, and vorinostat. The blue cluster (cluster 3) highlights research on epigenetic drugs targeting DNA methylation and histone modifications across various cancers, such as breast cancer, lung cancer, ovarian cancer, gastric cancer, colon cancer, pancreatic cancer, glioma, and others. The yellow cluster (cluster 4) delves into cellular or tumor states, addressing activation, growth, apoptosis, metastasis, tumor resistance, as well as the survival and prognosis of cancer patients. Lastly, the purple cluster (cluster 5) explores the intersection of epigenetics and immune regulation, covering topics like immunotherapy, antitumor immunity, tumor microenvironment, T-cells, regulatory T-cells, MHC class-I, dendritic cells, among others.

**TABLE 1:** Top 20 authors’ keywords of epigenetic therapy in oncology.

Rank	Key words	Records	Total links	Rank	Keywords	Records	Total links
1	Dna methylation	673	5,932	11	Apoptosis	162	1,439
2	Epigenetic therapy	550	4,752	12	Valproic acid	128	1,234
3	Epigenetics	445	4,074	13	Tumor-suppressor genes	116	1,140
4	Cancer	465	4,066	14	Cells	151	1,136
5	Expression	419	3,385	15	Decitabine	118	1,131
6	Methylation	278	2,410	16	Therapy	129	1,131
7	Gene-expression	277	2,264	17	Epigenetic drugs	120	1,082
8	Histone deacetylase inhibitors	231	2,116	18	Chromatin	120	1,029
9	Histone deacetylase inhibitor	216	2,063	19	Gene	123	1,027
10	Acute myeloid-leukemia	180	1,709	20	Hdac inhibitors	104	1,006

### 3.3 Evolution and burst of knowledge base

Co-citation bursts signify periods of rapid fluctuations in citation frequency for references within academic literature. The top 25 burst documents, delineated based on their initiation and cessation dates, are presented in sequential order (Figure 2C). Among the top 20 most cited works, 5 were articles while 15 were reviews. Notably, the document exhibiting the highest burst intensity, published in 2015, elucidated that DNA methyltransferase inhibitors (DNMTis) augment immune signaling in cancer through the viral defense pathway (Chiappinelli et al., 2015). Subsequently, the second-ranked study illustrated that low-dose 5-AZA-CdR targets colorectal cancer-initiating cells (CICs) by inducing viral mimicry, potentially via dsRNAs from endogenous retroviral elements. This induction activates the MDA5/MAVS RNA sensing pathway, followed by the activation of IRF7 (Roulois et al., 2015). Moreover, a detailed account of a phase I/II trial in recurrent metastatic non-small cell lung cancer patients was provided in another article. The trial combined azacitidine and entinostat (DNA methyltransferase and histone deacetylase inhibitors) for epigenetic therapy, resulting in a median overall survival of 6.4 months (95% CI 3.8–9.2), showcasing superiority over existing treatments (Juergens et al., 2011).The most pronounced co-citation burst within academic literature in the past 5 years was attributed to a review published in 2019. This review encapsulated the aberrant functions of epigenetic enzymes in DNA methylation, histone acetylation, and histone methylation during tumor progression. It underscored the research advancements in epigenetic enzyme inhibitors or targeted drugs (Cheng et al., 2019).

Utilizing VOSviewer, references have been categorized into five distinct color-coded clusters, where the thickness of links signifies the strength of collaboration as measured by Total Link Strength (TLS) (Figure 2D). Notably, the most robust TLS is associated with a review co-authored by Peter A. Jones and Stephen B. Baylin. This review succinctly outlines the impact of epigenetic alterations on the initial phases of tumorigenesis, delving into the functions of stem/progenitor cells while also highlighting the increasing relevance of these advancements in the realm of cancer management strategies (Jones and Baylin, 2007).

### 3.4 Trends of themes

**FIGURE 3:**
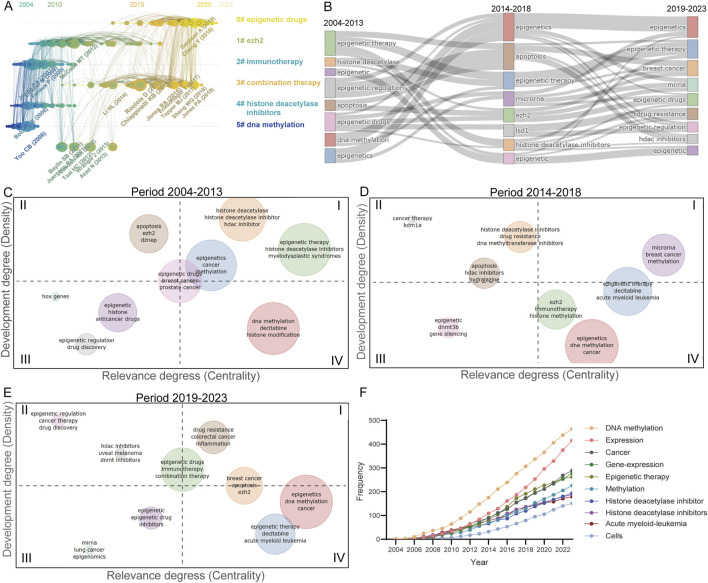
Trends in publications within the epigenetic therapy field in oncology. **(A)** Co-citation analysis of references over time. Nodes are scaled based on the number of co-citations, while the thickness and color of the node rings indicate the citation count per year. Nodes with rings signify high betweenness centrality, crucial for linking conceptual clusters across different time frames. The connections between references are depicted by link density, with each year assigned a distinct color. **(B)** Evolution of publication themes over the past two decades. **(C–E)** Thematic maps for the periods 2004–2013, 2014–2018, and 2019–2023. The thematic maps are segmented into four quadrants: Quadrant I: Motor themes with high density and centrality. II: Niche themes characterized by high density but low centrality. III: Emerging or declining themes with low density and centrality. IV: Basic themes with low density but high centrality. **(F)** Shifts in high-frequency keywords across time.

The aggregation of references within scholarly publications serves as a reservoir of scientific knowledge. By conducting co-citation analysis on these references, we can depict them in a chronological timeline format. The Co-citation Timeline illustrates six primary clusters discerned through co-citation analysis (Figure 3A). During the period spanning 2004 to 2013, the co-cited references predominantly centered around topics such as DNA methylation, histone deacetylase inhibitors, immunotherapy, and EZH2, reflecting the initial investigations into tumor epigenetic therapy. Subsequently, from approximately 2014–2023, the co-cited references primarily focused on epigenetic drugs and combination therapy, indicating current research focal hotspots in this field.

Thematic evolution diagrams and topic maps were created to elucidate the trends across various themes within the scholarly discourse. The progression of themes spanning the periods of 2004–2013, 2014–2018, and 2019–2023 has been visualized through the implementation of Sankey diagrams (Figure 3B). Topic maps, leveraging measures of centrality and density, have been employed to delineate the thematic evolution over distinct time frames (Figures 3C–E). Notably, during the interval of 2004–2013, themes such as “histone deacetylase,” “histone deacetylase inhibitors,” and “HDAC inhibitor” exhibited pronounced centrality and density. Transitioning from 2004–2013 to 2014–2018, there was a substantial escalation in both the centrality and density of breast cancer themes, underscoring the growing significance and advancements in the realm of epigenetic inhibitors for breast cancer therapeutics. A notable shift occurred in the period from 2019 to 2023, with the emergence of new topics like “drug resistance,” “immunotherapy,” and “combination therapy,” characterized by elevated centrality and density, signifying their rapid ascension as pivotal subjects of study. Furthermore, an analysis of high-frequency keywords and their temporal variations has been conducted (Figure 3F).

### 3.5 Analysis of publications and journals

Table 2 presents the details of the top 10 most cited publications in this work. The publication that stands out with the highest number of citations is a review article authored by Egger et al. (2004), published in 2004. This article delves into the landscape of human diseases within the realm of epigenetics and explores the potential of epigenetic therapy. It encapsulates discussions on epigenetic diseases, therapeutic interventions, and offers a forward-looking perspective (Egger et al., 2004). Another noteworthy mention is a review penned by Mark A. Dawson and Tony Kouzarides, boasting the highest average yearly citation rate. This review elucidates the foundational concepts underpinning epigenetic pathways, encompassing DNA methylation, histone modification, nucleosome remodeling, and RNA-mediated regulatory mechanisms. It underscores the evidence suggesting that dysregulation of these pathways could culminate in carcinogenesis (Dawson and Kouzarides, 2012).

**TABLE 2:** The top 10 most cited research papers.

Rank	First author	Journal	Year	Global citations	Citation frequency per year	Title
1	Egger G	Nature	2004	2,344	117.2	Epigenetics in human disease and prospects for epigenetic therapy
2	Dawson MA	Cell	2012	2,212	184.33	Cancer epigenetics: from mechanism to therapy
3	Sharma S	Carcinogenes	2010	1813	129.5	Epigenetics in cancer
4	Dawson MA	Nature	2011	1,204	92.62	Inhibition of BET recruitment to chromatin as an effective treatment for MLL-fusion leukaemia
5	Yoo CB	Nat Rev Drug Discov	2006	1,054	58.56	Epigenetic therapy of cancer: past, present and future
6	Rodríguez-paredes M	Nat Med	2011	917	70.54	Cancer epigenetics reaches mainstream oncology
7	Jones PA	Nat Rev Genet	2016	784	98	Targeting the cancer epigenome for therapy
8	Kantarjian H	Blood	2007	552	32.47	Results of a randomized study of 3 schedules of low-dose decitabine in higher-risk myelodysplastic syndrome and chronic myelomonocytic leukemia
9	Wu Q	Cancer Lett	2014	546	54.6	Multi-drug resistance in cancer chemotherapeutics: mechanisms and lab approaches
10	Yang H	Leukemia	2014	537	53.7	Expression of PD-L1, PD-L2, PD-1 and CTLA4 in myelodysplastic syndromes is enhanced by treatment with hypomethylating agents

**FIGURE 4:**
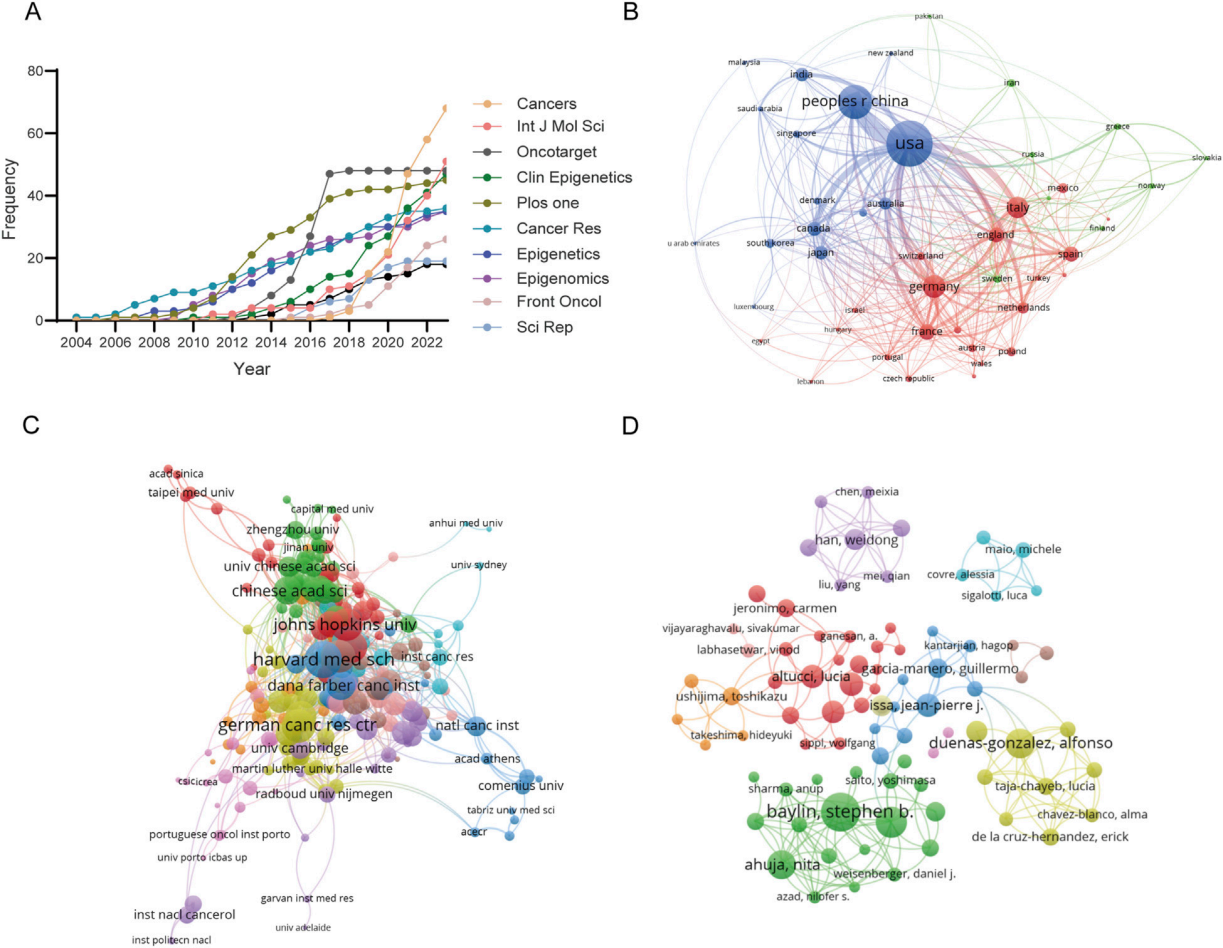
Attribution sources and collaboration networks in epigenetic therapy within oncology. **(A)** Analysis of the cumulative growth pattern of publications in the top 10 productive journals. **(B)** Visualization of co-authorship relationships among countries/regions. Node size represents the number of publications, while the thickness and length of links between nodes signify the strength and relevance of connections. **(C)** Co-authorship relationships among institutions. **(D)** Co-authorship relationships among authors.

All articles are distributed among 109 different journals. The graphical representation in Figure 4A and the detailed tabulation in Table 3 delineate the cumulative growth trajectory of yearly publications and furnish information into the top 10 journals exhibiting high productivity. Notably, the journals “*Cancers*,” “*International Journal of Molecular Sciences*,” and “*Oncotarget*” emerge as the leading triad in publication volume, with “Cancers” exhibiting the highest productivity by issuing 68 articles. In terms of citation frequency, the preeminent journals are “*Nature*,” “*Cancer Research*,” and “*Carcinogenesis*.” Delving into the metric of total link strength, the standout journals are “*Cancers*,” “*Epigenomics*,” and “*Clinical Epigenetics*.” Evidently, “*Cancers*” distinguishes itself as the most prolific publication platform, while “*Nature*” garners acclaim as the most influential journal, boasting the highest average citation.

**TABLE 3:** The top 10 productive journals related to epigenetic therapy in oncology.

Rank	Name	Country/region	Number of publications	% Of total publication	Average citation per publication	Total link strength
1	Cancers	United States	68	3.16	18.9	26,833
2	International Journal of Molecular Sciences	Switzerland	51	2.37	24.7	17,838
3	Oncotarget	United States	48	2.23	44.5	12,792
4	Clinical Epigenetics	Germany	47	2.18	43.6	22,008
5	Plos One	United States	45	2.09	31.2	15,532
6	Cancer Research	United States	36	1.67	89.8	13,353
7	Epigenetics	United States	35	1.63	29.7	14,858
8	Epigenomics	England	35	1.63	25.9	22,493
9	Frontiers in Oncology	Switzerland	26	1.21	14.9	10,040
10	Scientific Reports	England	19	0.88	21.6	5,478

### 3.6 Attribution and collaboration of countries/regions, authors and institutions

A total of 48 countries/regions have contributed to this research field, and the publications have been meticulously compiled and classified by country/region, as delineated in Table 4. Leading the pack in terms of publication count is the United States, boasting a substantial 745 publications. Noteworthy is Italy, which stands out for its remarkable publication density per trillion GDP, registering an impressive 88.79. The collaborative efforts among these countries/regions are vividly depicted in Figure 4B, where they are clustered and color-coded for clarity. The strength of collaboration, as quantified by TLS, is visually represented by the thickness of the connecting lines. At the forefront of collaboration are the United States (TLS = 409), followed by China (TLS = 191) and Germany (TLS = 190). The visual representation highlights three main clusters: the blue cluster, centered around the United States and China, features collaboration with Canada, Japan, India, and South Korea. Meanwhile, the red cluster is anchored by Germany, France, Italy, and the United Kingdom. The green cluster predominantly comprises European nations, with notable hubs in Switzerland, Norway, and Scotland. Furthermore, institutional collaborations are categorized into ten distinct clusters, as depicted in Figure 4C. Notably, Harvard Medical School emerges as a prominent collaborator with the highest TLS score of 73.

**TABLE 4:** The top 10 productive countries/regions in the field of epigenetic therapy in oncology.

Rank	Country/region	Numbers of publications	Publications 10 million people^a^	Publications per trillion GDP^a^	Numbers of citations	Average citations per publication	Co-authorship total link strength
1	UNITED STATES	745	22.35	29.28	46,329	62.19	409
2	PEOPLES R CHINA	435	3.08	24.22	12,070	27.75	191
3	GERMANY	208	24.82	50.95	8,671	41.69	190
4	ITALY	182	30.88	88.79	6,096	33.49	118
5	FRANCE	103	15.15	37.06	5,815	56.46	145
6	SPAIN	99	20.72	69.83	6,611	66.78	83
7	ENGLAND	96	14.33	31.08	7,532	78.46	147
8	CANADA	93	23.89	43.03	4,373	47.02	114
9	INDIA	80	0.56	23.41	2,169	27.11	49
10	JAPAN	78	6.23	18.33	2,883	36.96	55

^a^ Calculations based on 2022 population and GDP data from world bank (https://databank.worldbank.org/). GDP is calculated using GDP (current US$).

Table 5 presents a concise overview of the top 10 most prolific authors in the field. The collaborative dynamics among researchers are visually represented in Figure 4D, delineated into 11 distinct clusters. Noteworthy contributions include Peter A. Jones, who boasts the highest citation count of 5,085. Additionally, Stephen B. Baylin emerges as a leader in publications, with 27 to his credit, as well as possessing impressive h-index (26) and g-index (31) scores. Meanwhile, Table 6 meticulously details the top ten academic institutions by publication output. MD Anderson Cancer Center at the University of Texas stands out with the highest number of publications (57). The University of Southern California leads in both total citations (8,920) and average citations per publication (318.57), underscoring its significant impact in the field.

**TABLE 5:** The top 10 productive authors in the field of epigenetic therapy in oncology.

Rank	Author	Affliation	Numbers of publications	Numbers of citations	Average citation per publication	Co-authorship total link strength	H-index	G-index
1	Baylin, Stephen B	Johns Hopkins University	27	3,564	132	51	26	31
2	Jones, Peter A	University of Southern California	20	5,085	254.25	38	23	26
3	Duenas-gonzalez, Alfonso	Universidad Nacional Autonoma de Mexico	19	950	50	58	12	13
4	Ahuja, Nita	Yale School of Medicine	18	1,381	76.72	40	14	18
5	Issa, Jean-pierre j	Lewis Katz School of Medicine at Temple University	14	3,036	216.86	12	15	16
6	Altucci, Lucia	University of Campania “Luigi Vanvitelli”	14	819	58.5	16	15	16
7	Lübbert, Michael	University of Freiburg	14	565	40.36	4	17	21
8	Jung, Manfred	University of Freiburg	13	531	40.85	10	6	11
9	Esteller, Manel	University of Barcelona	12	2686	233.83	0	16	18
10	Han, Weidong	Chinese PLA General Hospital	12	394	32.83	40	10	12

**TABLE 6:** The top 10 productive institutions in the field of epigenetic therapy in oncology.

Rank	Institution	Country/region	Numbers of publications	Numbers of citations	Average citation per publication	Co-authorship total link strength
1	The University of Texas MD Anderson Cancer Center	United States	57	4,231	74.23	58
2	Johns Hopkins University	United States	48	3,926	81.793	56
3	Universidad Nacional Autonoma de Mexico	Mexico	37	1,445	39.053	16
4	National Cancer Institute	United States	34	2,581	75.913	55
5	University of Freiburg	Switzerland	34	1,589	46.74	35
6	Sun Yat-sen University	China	31	1,484	47.87	32
7	University of South California	United States	28	8,920	318.57	22
8	German Cancer Research Center	German	27	1,047	38.78	60
9	Shanghai jiao tong University	China	27	558	20.67	26
10	Chinese Academy of Sciences	China	25	747	29.88	42

## 4 Discussion

In recent decades, the field of epigenetics in biology has undergone a significant transformation, challenging longstanding traditional perspectives regarding the genetic code as the primary determinant of cellular gene function and the leading cause of human diseases (Sharma et al., 2010). Progress in cancer epigenetics has prompted the realization that genome packaging may be just as critical as the genome itself in regulating fundamental cellular processes essential for maintaining cell characteristics and triggering disease states such as cancer (Yoo and Jones, 2006; Baylin and Jones, 2011). The emergence of numerous drugs targeting specific enzymes involved in epigenetic regulation of gene expression has made the utilization of epigenetic targets an increasingly effective and valuable approach in chemotherapy and cancer chemoprevention (Yoo and Jones, 2006).

This study aims to explore the research focal points, knowledge base expansion, and trends in epigenetic inhibitors in tumors over the past two decades. An analysis of literature published from 2004 to 2023 in this field was conducted, and the findings are presented visually. After excluding studies that did not meet the selection criteria, our analysis covered 2,153 English-language papers published in 109 journals from 249 institutions across 48 countries/regions. This research offers a bibliometric analysis of studies on epigenetic therapies in oncology, with the goal of providing researchers with a comprehensive understanding of cancer epigenetic treatments.

Epigenetic therapies for cancer have garnered significant interest, evident from the expanding body of literature in this field. The milestone approval of the first epigenetic drug, azacitidine (AZA), by the FDA in 2004 marked a pivotal transition from theoretical exploration to practical application. A notable moment occurred in 2006 with the approval of decitabine for treating MDS, signifying a crucial advancement in the utilization of epigenetic drugs for cancer treatment. From 2004 to 2013, research literature extensively delved into the application of epigenetic inhibitors in hematologic malignancies, initial efficacy studies in solid tumors, and their combined use with other treatment modalities. Issa et al. demonstrated the substantial efficacy of low-dose extended exposure schedules of decitabine in refractory hematologic malignancies (Issa et al., 2004). Moreover, an international, multicenter, phase III clinical trial utilizing an open-label, parallel-group design revealed that azacitidine treatment significantly improved overall survival in high-risk MDS patients (Fenaux et al., 2009). McCabe et al. (2012) suggested that inhibiting EZH2 methyltransferase activity could be a promising strategy for treating diffuse large B-cell lymphoma and follicular lymphoma with EZH2 activating mutations. In addition to hematologic malignancies, research has initially explored the use of epigenetic inhibitors in solid tumors, such as non-small cell lung cancer (Juergens et al., 2011) and breast cancer (Tsai et al., 2012). Furthermore, research has emphasized the synergistic benefits of epigenetic drugs, whether used alone or in combination with chemotherapy, immunotherapy, or radiation therapy. These combined approaches not only enhance therapeutic efficacy but also help mitigate potential drug resistance (Bolden et al., 2006; Yoo and Jones, 2006; Dawson and Kouzarides, 2012).

In the past decade, scholarly research has further focused on investigating the efficacy of epigenetic inhibitors in treating solid tumors, including non-small cell lung cancer (Wrangle et al., 2013; Topper et al., 2017), breast cancer (Li et al., 2014), ovarian cancer (Li et al., 2014; Chiappinelli et al., 2015), and colorectal cancer (Li et al., 2014; Roulois et al., 2015). These studies have revealed the potential of epigenetic inhibitors to modulate immune induction pathways within tumors. For instance, in non-small cell lung cancer cell lines, the use of AZA has been shown to increase the expression of the inhibitory ligand PD-L1, resulting in the consistent downregulation of immune genes and PD-L1 expression in specific subsets of primary tumors. This finding suggests that combining epigenetic therapy with PD-1 pathway blockade could lead to a synergistic anti-tumor response (Wrangle et al., 2013). In ovarian cancer, DNA methyltransferase inhibitors (DNMTis) activate the viral defense pathway, thereby enhancing immune signaling in cancer cells (Chiappinelli et al., 2015). Similarly, in colorectal cancer, brief exposure to low doses of 5-AZA-CdR can induce dsRNA expression, activating the cytoplasmic pattern recognition receptor MDA5 and subsequently engaging downstream effectors MAVS and IRF7 to target colorectal cancer cells (Roulois et al., 2015). Moreover, in non-small cell lung cancer, the combined treatment of HDAC inhibitors and AZA has shown a significant anti-tumor response by inhibiting myc-driven cell proliferation and amplifying immune signals (Topper et al., 2017). Consequently, epigenetic inhibitors impact immune cells within the tumor microenvironment, synergizing with immunotherapy to enhance anti-tumor immune responses and improve clinical outcomes (Topper et al., 2017).

In the past 5 years, research on epigenetic therapy in oncology has witnessed a notable shift in focus towards themes such as “drug resistance,” “immunotherapy,” and “combination therapy,” marking an evolution in research priorities. This shift highlights a substantial increase in the attention and research intensity dedicated to these areas compared to the period spanning 2004 to 2018. Notably, targeted epigenetic therapy has gained wide acceptance in both preclinical and clinical trials for hematologic malignancies, signifying promising applications for treating solid tumors (Cheng et al., 2019).The utilization of epigenetic drugs, including demethylating compounds and HDAC inhibitors, has exhibited the ability to reactivate tumor suppressor genes and essential cellular functional genes by specifically targeting abnormal chromatin regions (Jones and Baylin, 2007). Consequently, the employment of these agents can expand the population of chemosensitive cells, thereby providing viable targets for alternative treatment modalities like chemotherapy, immunotherapy, or radiation therapy. Given the short-term impacts of demethylating agents and their role in restoring aberrant methylation patterns, combining epigenetic therapy with other interventions could potentially enhance treatment efficacy (Jones and Baylin, 2007). Moreover, strategies geared towards overcoming drug resistance and enhancing cancer cell sensitivity to multiple treatments show promise (Azad et al., 2013). Research findings strongly suggest that epigenetic therapy has the capacity to modulate tumor immune induction pathways, ultimately heightening tumor cell susceptibility to T-cell immune responses. Consequently, the concurrent application of epigenetic therapy and immune checkpoint blockade holds the potential for therapeutic advantages (Wrangle et al., 2013; Li et al., 2014; Chiappinelli et al., 2015; Topper et al., 2017).

China ranks second among the top ten most productive countries/regions, closely following the United States. Reflecting this national distribution, Chinese and American institutions dominate seven of the top ten positions, underscoring their significant contributions to the academic advancement of this field. Despite China’s substantial publication output, its average citation per paper lags significantly behind other countries, suggesting a dearth of highly referenced papers. The top ten academic journals collectively published 410 papers, constituting 19.04% of the total output. “*Cancers*” leads in the number of publications, followed by the “*International Journal of Molecular Sciences*” and “*Oncotarget,*” showcasing these journals’ keen interest in cancer epigenetic therapy research. The most cited articles were featured in “*Nature*.” Notably, among the top 20 most cited works, two originated from “*Nature*,” two from “*Cell*,” and three from “*Blood*,” indicating these influential journals’ propensity to publish high-caliber research in the future.

Epigenetic events play a pivotal role in both normal biological processes and tumorigenesis, with significant alterations in the epigenetic state commonly observed during cancer progression. This has led to the emergence of epigenomic targeted therapy as a promising avenue for cancer treatment. However, several critical issues warrant further discussion and resolution. While remarkable strides have been made in applying epigenetic therapy to hematologic malignancies, its effectiveness in solid tumors remains to be conclusively demonstrated. In preclinical models, compelling mechanistic evidence supports the notion that epigenetic agents can synergize with other anticancer drugs and combat treatment resistance. Nevertheless, the clinical efficacy of epigenetic agents tested in trials has been underwhelming thus far (Morel et al., 2020). Challenges arise from the limited tolerability of combinations involving epigenetic agents and cytotoxic therapies. Exploring strategies such as lower doses, sequential administration, and targeted delivery of epigenetic agents holds promise for enhancing the therapeutic index (Morel et al., 2020). Furthermore, the prolonged duration required for epigenetic reprogramming, in contrast to traditional chemotherapy, necessitates an understanding that the initial response to epigenetic therapy may not be immediately apparent. Consequently, the conventional Response Evaluation Criteria in Solid Tumors (RECIST) criteria, typically applied within 6–8 weeks to assess clinical response, may not be optimal for monitoring epigenetic therapy in clinical trials (Azad et al., 2013). Continued treatment may be warranted for clinically stable patients undergoing epigenetic therapy.

## 5 Future research directions

Overall, significant progress has been made in the discovery of epigenetic drugs over the past few decades. While certain epigenetic therapies have demonstrated favorable clinical outcomes and received regulatory approval for hematologic malignancies, achieving and maintaining therapeutic effects in solid tumors continues to pose challenges (Feng and De Carvalho, 2022). Results from preclinical studies and clinical trials underscore the potential efficacy of combining epigenetic inhibitors with chemotherapy or immunotherapy. Further exploration of the sequencing of combination treatments, as well as optimization of trial designs and dosing regimens, may be necessary to enhance clinical efficacy. Moreover, the sample sizes of the clinical trials conducted thus far have been relatively small, and there is a lack of effective predictive biomarkers. Research into the potential mechanisms and biomarkers associated with combination therapy can deepen our understanding and inform future treatment strategies.

Additionally, it has been demonstrated that epigenetics affects tumor immunogenicity and the immune cells involved in anti-tumor responses (Yang et al., 2023). Studies suggest that developing therapies targeting epigenetic modification pathways can bolster the efficacy of immunotherapy. However, specifically targeting epigenetics without inducing severe toxicity remains a substantial challenge. Consequently, comprehending the mechanisms of epigenetic modifications and mastering their control methods is an area deserving further investigation. For example, precisely targeting epigenetic modification sites could significantly reduce off-target effects and other adverse reactions.

Finally, both epigenetics and novel immunotherapies are emerging tools in clinical practice that necessitate more research to identify and develop reliable epigenetic biomarkers (Villanueva et al., 2020). These candidate biomarkers may provide a theoretical basis for patient stratification and precision medicine, thereby maximizing the chances of therapeutic success while minimizing unintended consequences. By leveraging this understanding, new generations of epigenetic drugs suitable for use in combination with immunotherapy may be developed.

## 6 Limitation

This study employs bibliometric methods to analyze the development trends and potential research frontiers in epigenetic therapy for tumors. Its goal is to provide a historical perspective for future research and to highlight areas that warrant further investigation. However, certain limitations should be acknowledged in this survey. The WOSCC database provides standardized and comprehensive bibliometric records, making it one of the most reliable data sources available (Merigó and Núñez, 2016). Consequently, our search was confined to this database, which may have resulted in the exclusion of some studies outside its scope. Furthermore, it is important to recognize that the impact of a paper can be influenced by its publication date; thus, some recently published high-quality articles might be overlooked due to their low citation frequency. Lastly, this study involves numerous authors, some of whom may have changed names or collaborated across multiple institutions. While we have meticulously reviewed the process, some errors are inevitable. Nevertheless, these limitations are unlikely to affect the fundamental trends presented in this article. The visualized bibliometric analysis can still effectively assist researchers in understanding the hotspots and emerging trends in tumor epigenetic therapy research.

## 7 Conclusion

Over the past two decades, the significance of epigenetic therapy in cancer research has increasingly come to prominence, with the field of epigenetics rapidly transforming approaches to cancer treatment. The United States occupies a critical position in the study of epigenetic therapies for tumors, while China, despite having published a substantial number of related papers, still exerts limited influence. Currently, the focus within the field of epigenetic therapy mainly revolves around the “combination therapy” of epigenetic drugs. Both genetic mutations and epigenetic abnormalities contribute to cancer progression; thus, integrating traditional carcinogenic pathways with epigenetic therapy may provide effective solutions for treating solid tumors. Future research should explore the sequencing of combination therapies and optimize trial designs and dosing regimens to enhance clinical efficacy. Additionally, it is vital to investigate the potential mechanisms and biomarkers associated with combination therapies and to develop new generations of epigenetic drugs that precisely target epigenetic modification sites. These efforts are anticipated to advance the application of epigenetic therapy in oncology, ultimately aiding patients in achieving better treatment outcomes.

## Data Availability

The original contributions presented in the study are included in the article/Supplementary Material, further inquiries can be directed to the corresponding author.

## Abbreviations

MDS, Myelodysplastic syndromes; HDAC, Histone deacetylase; WoSCC, Web of Science Core Collection; DNMTis, DNA methyltransferase inhibitors; TLS, Total Link Strength; AZA, Azacitidine; AML, acute myeloid leukemia; CML, chronic myeloid leukemia.
